# Prevalence of chronic kidney disease in population-based studies: Systematic review

**DOI:** 10.1186/1471-2458-8-117

**Published:** 2008-04-11

**Authors:** Qiu-Li Zhang, Dietrich Rothenbacher

**Affiliations:** 1 Division of Clinical Epidemiology and Aging Research, German Cancer Research Center, Heidelberg, Germany

## Abstract

**Background:**

Chronic kidney disease (CKD) is becoming a major public health problem worldwide. This article reviews the published evidence of prevalence of CKD in population-based study samples that used the standardized definition from the Kidney Disease Outcomes Quality Initiative of the National Kidney Foundation (K/DOQI) practice guideline, and particularly focus on performance of serum-creatinine based equations for GFR estimation. We provide a summary of available data about the burden of CKD in various populations.

**Methods:**

We performed a systematic review of available published data in MEDLINE. A combination of various keywords relevant to CKD was used in this research. Related data of included studies were extracted in a systematic way.

**Results:**

A total of 26 studies were included in this review. The studies were conducted in different populations, and the number of study participants ranged from 237 to 65181. The median prevalence of CKD was 7.2% in persons aged 30 years or older. In persons aged 64 years or older prevalence of CKD varied from 23.4% to 35.8%. Importantly, the prevalence of CKD strongly depended on which estimating equations were used. The Modification of Diet in Renal Disease Study (MDRD) equation was likely to be preferred in recent epidemiological studies compared to the adjusted Cockcroft-Gault (CG) equation.

**Conclusion:**

Worldwide, CKD is becoming a common disease in the general population. Accurately detecting CKD in special groups remains inadequate, particularly among elderly persons, females or other ethnic groups such as Asians.

## Background

Chronic kidney disease (CKD) is becoming a major public health problem worldwide. The current burden of disease might due to a change of the underlying pathogenicity of CKD. Glomerulonephritis was the one of the leading causes of kidney disease several decades ago. Nowadays, infections have become a less important cause for kidney disease, at least in the western world [1]. Moreover, current evidence suggests that hypertension and diabetes are the two major causes of kidney disease worldwide [2,3]. Given the pathogenic progression of kidney disease, patients with CKD are at high risk for progression to the end stage renal disease (ESRD) – a condition requiring dialysis or kidney transplantation to maintain patients' long-term survival. In 2001, the average annual cost for maintenance of ESRD therapy was between US $70 and $75 billion worldwide excluding kidney transplantation, and the predicted number of ESRD patients will reach over 2 million in 2010 [4]. The enormous costs of treatment lead to a large burden for the health care systems, particularly in developing countries.

In addition, CKD has a complicated interrelationship with other diseases [5]. Recent studies have reported that CKD is an independent risk factor for cardiovascular disease (CVD) [6]. Therefore, kidney dysfunction should be an additional target for intervention and prevention of CVD [7]. In 2003, the American Heart Association (AHA) stated that persons with CKD should be regarded as the highest risk group for subsequent CVD [8].

Due to the asymptomatic nature of this disease, CKD is not frequently detected until its later progress, resulting in lost opportunities for prevention. Progress to kidney failure or other adverse outcomes could be prevented or delayed through early detection and treatment of CKD [9,10].

Currently, large efforts have been made to a better detection of progressive kidney disease. In 2002 the Kidney Disease Outcomes Quality Initiative (K/DOQI) of the National Kidney Foundation (NKF) developed a practice guideline for CKD [11]. According to this guideline, CKD is defined as either kidney damage or glomerular filtration rate (GFR) below 60 ml/min/1.73 m^2 ^for three or more months with or without evidence of kidney damage, irrespective of the cause [12]. GFR is estimated by serum creatinine based on equations rather than on direct measurements. Several equations have been developed and the most frequently used ones are the Cockcroft-Gault (CG) equation and the Modification of Diet in Renal Disease Study (MDRD) equation. Both equations are currently considered to be the best methods to estimate GFR for adults in epidemiologic studies [13-15].

A number of epidemiologic studies assessed the prevalence of CKD in different populations and used different equations to estimate kidney function. This article reviews the recently published data on the prevalence of CKD in population-based study samples that used the standardized definition from K/DOQI practice guideline, particularly focuses on performance of estimating equations for GFR such as the MDRD equation and the CG equation, and provides a summary for the burden of CKD.

## Methods

### Search strategy

A systematic literature search was conducted in the MEDLINE database (US National Library of Medicine, Bethesda, Maryland) to identify all potentially relevant publications before July 2006. The following words were used for this search: "chronic kidney (or renal) disease", "kidney (or renal) disease", "kidney (or renal) dysfunction", "decreased kidney (renal) function", "glomerular filtration rate", "Cockcroft-Gault equation", "MDRD equation", "prevalence", and "population" or "community". Reference lists of primary original studies and review articles were also checked whether any further related articles could be found (cross-references).

### Study selection

Inclusion criteria were designed to find studies that reported the prevalence of CKD in the general population. Studies were included when carried out in a representative sample of the general population, and when the definition of CKD was based on the K/DOQI practice guideline. We excluded studies with a sample size of less than 50 participants, studies without GFR estimation by serum creatinine-based equations, and studies that provided only serum creatinine concentration. Information published only in abstract form was not included, as the abstracts usually do not provide all necessary information to evaluate the quality of a study.

### Definition of chronic kidney disease

We used the definition of CKD from the K/DOQI practice guideline that was published in 2002 by the National Kidney Foundation (NKF). CKD was defined as CrCl or GFR less than 60 ml/min/1.73 m^2 ^[11,12].

Although CrCl is different from GFR, it is commonly used as an estimation value in practice after adjusting for body surface area (BSA); therefore, we also used CrCl to assess the outcome, and compared CrCl with GFR.

The two equations were defined in the following way:

Simplified MDRD equation [15,16]: GFR (ml/min/1.73 m^2^) = 186.3 * (serum creatinine)^-1.154 ^* (age)^-0.203 ^* (0.742 if female) * (1.21 if African American).

CG/BSA equation [14]: Creatinine Clearance (ml/min) = (140 – age)/(serum creatinine) * (weight/72) * (0.85 if female), which is further standardized for body surface area (BSA) according to the Dubois and Dubois formula [17]: BSA (m^2^) = 0.20247 * height (m) ^0.725 ^* weight (kg) ^0.425^. Creatinine Clearance is also expressed as ml/min/1.73 m^2^.

Serum creatinine is measured in mg/dl, age in years, weight in kg, and GFR is expressed as ml/min/1.73 m^2^.

### Data extraction

Two investigators reviewed each paper independently. Discrepancies were discussed and agreement was achieved by consensus. When possible, the prevalence of CKD was extracted as an overall value and stratified by estimating equations, by age categories and by gender. In some included studies, the prevalence of CKD was not directly stated but available from published figures or calculated from other available information of the articles. We presented the prevalence of CKD based on GFR estimation only, although both GFR estimation and proteinuria were reported in some of the included studies.

## Results

### Study characteristics

**Figure 1:**
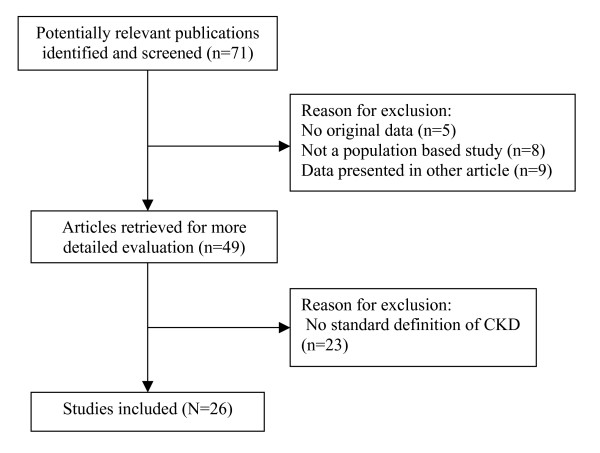
Flow diagram of studies.

We retrieved 71 publications of potential interest for this review, of which 45 studies were excluded (Fig 1). Finally, we identified 26 articles fulfilling our inclusion criteria [18-43]. Ten studies were from America (Table 1) [18-27], 8 studies were from Europe (Table 2) [28-35], and 8 were from Asia and Australia (Table 3) [36-43]. The number of participants in the included studies ranged from 237 to 65181 [41,30]. The subjects of included studies were all adults (≥ 18 years old); 4 studies assessed the prevalence of CKD among elderly persons over 63 years old [23,24,26,35], and 10 studies among middle and old-aged persons (≥ 30 years old) [25,27,28,34,37-40,42,43]. Two studies were based on the data from the NHANES III study, but used different time periods (1988–1994 and 1999–2000); both of these were included in this review [20,21].

**Table 1:** Prevalence of chronic kidney disease (CKD) in population-based studies from America

			Prevalence of CKD				
			
Author [ref.] year	Country	Study population, study design, number of participants, response, age, gender	MDRD equation			CG/BSA equation	
				
Amato et al. [18] 2005	Mexico	Randomly selected participants from primary care facilities in a large city, cross-sectional study, N = 3564, response NR, aged >18 yrs., gender NR.	NR			Overall: 8.5%	
				
Brown et al. [19] 2005	USA	Participants of the Kidney Early Evaluation Program (KEEP) in 33 states, cross-sectional study, N = 6071, response NR, aged 18–101 yrs. (mean age: 52 yrs.), 32% males.	Overall: 15.6% Men: 14.4%, Women: 16.2%			NR	
			Age (yrs.)	Men	Women		
			18–30	2.4%	2.7%		
			31–45	5.4%	6.4%		
			46–60	9.5%	11.5%		
			61–75	24.3%	29.9%		
			76+	45.6%	45.0%		
				
Coresh et al. [20] 2005	USA	Participants of the Third National Health and Nutrition Examination Survey (NHANES III, 1999–2000), cross-sectional study, N = 4101, response NR, aged ≥ 20 yrs., 47.7% males.	Overall: 3.8% Men: 2.7%, women: 4.8% White: 4.2%, African American: 3.4%, Mexican American: 1.2%, other: 3.2%			NR	
			Age (yrs.)				
			20–39	0.5%			
			40–59	1.5%			
			60–69	6.2%			
			70+	23.1%			
				
Coresh et al. [21] 2003	USA	Participants of the Third National Health and Nutrition Examination Survey (NHANES III 1988–1994), cross-sectional study, N = 15600, response NR, aged ≥ 20 yrs., 47% males.	Overall: 4.5% Men: 3.6%, Women: 5.3% White: 5.0%, African American: 3.3%, Mexican American: 1.0%, other: 2.2%			Overall: 7.0% Men: 6.3%, Women: 7.7% White: 7.5%, African American: 7.8%, Mexican American: 1.8%, other: 4.1%	
			Age (yrs.)			Age (yrs.)	
			20–39	0.2%		20–39	-
			40–59	1.8%		40–59	0.8%
			60–69	7.6%		60–69	10.5%
			70+	24.9%		70+	^4^^9.2%^
				
Fox et al. [22] 2006	USA	Participants from the sixth examination of the Framingham Offspring Study, cohort study, N = 3047, response NR, mean age: 59 yrs., 48% males.	Overall: 8.6%			NR	
				
Garg et al. [23] 2004	Canada	Participants from long-term care facilities in the elderly, retrospective cross-sectional study, N = 9931, response 85%, aged ≥ 65 yrs. (mean age: 82 yrs.), 26% males.	Overall: 35.7% Men: 27.1%, Women: 38.8%			NR	
			Age (yrs.)	Men	Women		
			65–69	9.2%	22.8%		
			70–74	14.9%	23.8%		
			75–79	21.7%	29.2%		
			80–84	27.5%	35.2%		
			85–89	32.8%	41.9%		
			90–94	40.5%	47.3%		
			95+	37.8%	50.7%		
				
Hemmelgarn et al. [24] 2006	Canada	Participants from community-dwelling elderly in Calgary Health region, cohort study, N = 10184, response NR, aged ≥ 66 yrs., 42.6% males.	Overall: 35.4% Men: 32%, Women: 38.2%			NR	
				
Kramer et al. [25] 2005	USA	Participants of the Dallas Heart Study, cross-sectional survey, N = 2660, response NR, aged 30–65 yrs.(mean age: 43.9 yrs.), 49.5% males.	Overall: 1.5%			NR	
				
Manjunath et al. [26] 2003	USA	Participants of the Cardiovascular Health Study (CHS), cohort study, N = 4893, response NR, aged ≥ 65 yrs. (mean age: 75.4 yrs.), 44.5% males.	Overall: 23.4% Men: 25%, Women: 22.2%			NR	
				
McClellan et al. [27] 2006	USA	Randomly selected participants of the Reasons for Geographic and Racial Differences in Stroke (REGARDS) study, cohort study, N = 20667, response NR, aged ≥ 45 yrs., 48.8% males.	Overall: 43.3% Men: 38.9%, Women: 47.5% White: 49.9%, African American: 33.7%			NR	
			Age (yrs.)				
			45–54	19.3%			
			55–64	31.6%			
			65–74	51.3%			
			75–84	62.7%			
			85+	71.0%			

MDRD: simplified equation of the Modification of Diet in Renal Disease Study.

CG/BSA: Cockcroft-Gault formula adjusted by Body Surface Area.

GFR: Glomerular Filtration Rate. NR: Not reported.

**Table 2:** Prevalence of chronic kidney disease (CKD) in population-based studies from Europe

			Prevalence of CKD				
			
Author [ref.] year	Country	Study population, study design, number of participants, response, age, gender	MDRD equation			CG/BSA equation	
				
Brugts et al. [28] 2005	Netherlands	Participants of the Rotterdam Study, prospective cohort study, N = 4484, response 78%, aged ≥ 55 yrs. (mean age: 69.6 yrs.), 36.3% males.	NR			Overall: 44.9%	
				
Cirillo et al. [29] 2006	Italy	Participants from central Italy, cross-sectional study, N = 4574, response NR, aged 18–95 yrs., 45.5% males.	Overall: 6.4% Men: 6.5%, Women: 6.2%			NR	
			Age (yrs.)	Men	Women		
			18–44	0.6%	1.3%		
			45–54	2.6%	1.3%		
			55–64	7.3%	5.4%		
			65–74	15.0%	11.0%		
			75+	34.5%	31.6%		
				
Hallan et al. [30] 2006	Norway	Participants of the second Health Survey of Nord-Trondelag County (HUNT II), cross-sectional study, N = 65181, response 70.4%, aged ≥ 20 yrs. (mean age: 50.2 yrs.), 46.8% men.	Overall: 4.7% Men: 3.6%, Women: 5.7%			NR	
			Age (yrs.)				
			20–39	0.2%			
			30–59	1.4%			
			60–69	6.3%			
			70+	18.6%			
				
Nitsch et al. [31] 2006	Switzerland	Participants of the Swiss SAPALDIA Study, random sample, cross-sectional study, N = 6317, response NR, aged ≥ 18 yrs., 49% males.	Overall: 8.1% Men: 4.5%, Women: 11.5%			NR	
			Age (yrs.)	Men	Women		
			< 55	1.1%	7.9%		
			55–65	7.1%	23.5%		
			66+	12.9%	35.9%		
				
Otero et al. [32] 2005	Spain	Randomly selected participants of the Estudio Epidemioloógic o de la Insuficiencia Renal en Espana (EPIRCE) pilot study, cross-sectional study, N = 237, response NR, aged ≥ 20 yrs. (mean age: 49.58 yrs.), 42.6% males.	Overall: 5.1%			NR	
				
Verhave et al. [33] 2004	Netherlands	Participants of the Prevention of Renal and Vascular End-stage Disease Study, cohort study, N = 6022, response NR, aged 28–75 yrs. (mean age: 48 yrs.), 51.5% males.	NR			Overall: 4.2%	
				
Viktorsdottir et al. [34] 2005	Iceland	Participants of the Reykijavik Heart Study, cross-sectional study. N = 19256, response NR, aged 33–85 yrs., 48% males.	Overall: 7.2% Men: 3.7%, Women: 10.9%			Overall: 24.7% Men: 19%, Women: 30%	
			Age (yrs.)	Men	Women		
			35–39	0.8%	2.2%		
			40–44	1.1%	3.0%		
			45–49	1.3%	4.4%		
			50–54	2.3%	6.3%		
			55–59	2.4%	7.6%		
			60–64	5.2%	11.7%		
			65–69	13.5%	36.1%		
			70–74	17.0%	38.1%		
			75–79	19.5%	35.3%		
			80+	24.5%	53.1%		
				
Wasen et al. [35] 2004	Finland	Participants from elderly residents in a community, cross-sectional study, N = 1246, response 83%, age 64–100 yrs. (mean age: 74 yrs.), 42% males.	Overall: 35.8%			Overall: 58.5%	

MDRD: simplified equation of the Modification of Diet in Renal Disease Study.

CG/BSA: Cockcroft-Gault formula adjusted by Body Surface Area.

GFR: Glomerular Filtration Rate.

NR: Not reported.

**Table 3:** Prevalence of chronic kidney disease (CKD) in population-based studies from Asia and Australia

			Prevalence of CKD					
			
Author [ref.] year	Country	Study population, study design, number of participants, response, age, gender	MDRD equation			CG/BSA equation		
				
Chadban et al. [36] 2003	Australia	Randomly selected participants of the Australian Diabetes, Obesity and Lifestyle Study (AusDiab), cross-sectional survey, N = 11247, response 89.1%, aged 25 ≥ yrs., gender NR.	NR			Overall: 11.2% Men: 9.3%, Women: 13.0%		
						Age (yrs.)	Men	Women
						25–44	-	0
						45–64	1.8%	3.2%
						65+	51.8%	57.2%
				
Chen et al. [37] 2005	China	Participants of the International Collaborative Study of Cardiovascular Disease in Asia (InterASIA), random sample, cross-sectional study, N = 15540, response 83.3%, aged 35–74 yrs., 48.5% males.	Overall: 2.5% Men: 1.3%, Women: 3.8%			Overall: 20.4%		
			Age (yrs.)	Men	Women			
			35–44	0.2%	1.2%			
			45–54	0.7%	2.7%			
			55–64	1.6%	6.4%			
			65–74	5.8%	10.4%			
				
Domrongkitchaiporn et al. [38] 2005	Thailand	Participants of the Electricity Generating Authority of Thailand (EGAT) study, employees sample, cross-sectional study, N = 2967, response NR, aged 35–55 yrs., 76% males.	Overall: 6.8%	NR				
				
Konta et al. [39] 2006	Japan	Participants of the Molecular Epidemiological Study, cross-sectional survey, N = 2321, response NR, aged > 40 yrs. (mean age: 64 yrs.), 44:5% males.	NR			Overall: 28.8%		
				
Li et al. [40] 2006	China	Participants from residents in a district of a large city, cross-sectional survey, N = 2310, response NR, aged ≥ 40 yrs., 49.5% males.	Overall: 4.9% Men: 4.8%, Women: 5.0%			NR		
			Age (yrs.)	Men	Women			
			40–49	0	0.4%			
			50–59	1.5%	2.5%			
			60–69	4.4%	5.8%			
			70+	10.6%	12.9%			
				
McDonald et al. [41] 2003	Australia	Participants from a costal aboriginal community, cross-sectional study, N = 237, response NR, aged ≥ 18 yrs., 133 males.	Overall: 12%			NR		
				
Ninomiya et al. [42] 2005	Japan	Participants from study of Cerebrovascular and Cardiovascular Diseases, prospective cohort study, N = 2634, response 80.7%, aged ≥ 40 yrs., 42.1% males.	Overall: 10.3% Men: 5.3%, Women: 13.8%			NR		
				
Shankar et al. [43] 2006	Singa pore	Participants from private census, cross-sectional study, N = 4898, response 81.1%, aged 43–86 yrs. (mean age: 62.3 yrs.), 44% males.	Overall: 6.6% Men: 7.1%, Women: 6.2%			NR		
			Age (yrs.)					
			43–59	1.8%				
			60–69	6.5%				
			70–79	11.5%				
			80+	21.8%				

MDRD: simplified equation of the Modification of Diet in Renal Disease Study.

CG/BSA: Cockcroft-Gault formula adjusted by Body Surface Area.

GFR: Glomerular Filtration Rate.

NR: Not reported.

We extracted the prevalence of CKD from 19 cross-sectional studies [18-21,23,25,29-32,34-41,43], and 7 studies that were based on a cohort study design [22,24,26-28,33,42]. The assessment of the prevalence of CKD was not the major study object in some of the included studies. Some mainly determined the association between CKD and other risk factors, such as cardiovascular diseases [18,22,25,26,31,32,41], diabetes [18], and life style factors [43], or assessed the difference between estimating equations for kidney function [35].

### Prevalence of CKD

#### Equation-specific prevalence of CKD

Overall, 17 studies used the MDRD equation [19,20,22-27,29-32,38,40-43], 5 studies used the CG/BSA equation [18,28,33,36,39] and 4 studies used both of them [21,34,35,37]. Eight of 9 studies were published in 2006 with GFR estimation by the MDRD equation. The studies estimated GFR with both of the MDRD and the CG/BSA equations among the same population, and all reported a higher prevalence of CKD with the CG/BSA equation than with the MDRD equation [21,34,35,37]. A study based on the NHANES III survey provided the age-specified prevalence with both equations [21]. The increase of the prevalence with age was notably greater in using the CG/BSA rather than the MDRD equation, particularly in persons aged 70 years or older. The differences in the prevalence of CKD equations seemed much stronger among elderly persons than among younger ones.

#### Age-specific prevalence of CKD

**Figure 2:**
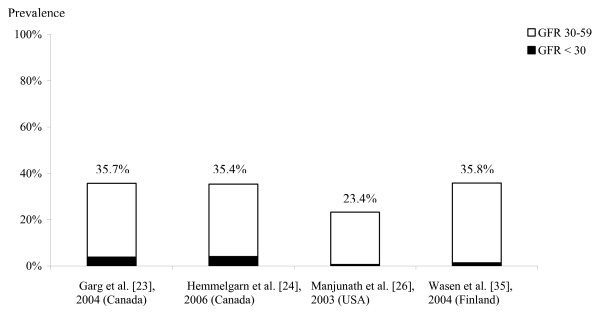
Prevalence of CKD stages in elderly persons (≥ 64 years) with CKD using the MDRD equation (GFR, ml/min/1.73 m^2^). The numbers over each bar represent whole prevalence of CKD (GFR < 60 ml/min/1.73 m^2^).

The prevalence of CKD varied strongly with age. Four studies were conducted among elderly persons (≥ 64 years) and showed a markedly high prevalence [23,24,26,35]. Manjunath et al. [26] measured serum samples from a community-based cohort study and found a prevalence of 23.4% by use of the MDRD equation for persons aged 65 years or older. In Canada, a cross-sectional study [23] showed a higher prevalence (35.7%) than the study of Manjunath et al. [26] in the USA. Meanwhile, Wasén et al. [35] used both MDRD and GC/BSA equations to estimate GFR in elderly persons in Finland and reported a much higher prevalence in using the CG/BSA equation (58.5%) than using the MDRD equation (35.8%). Another recent study from Canada reported an overall prevalence of 35.4% in elderly persons aged 66 years or older [24]. Figure 2 shows the prevalence of stages of CKD in studies conducted in population-based samples in the elderly with CKD.

Several studies were conducted in middle and old-aged persons (≥ 30 years old) [27,28,34,37-40,42,43]. The prevalences of CKD varied from 1.5% to 43.3% in different populations (median: 7.2%). The Dallas Heart Study [25], which included 2660 participants aged from 30 to 65 years old, reported the lowest prevalence of CKD by means of the MDRD equation (1.5%). The highest prevalence was reported from a cohort study among persons aged 45 years or older in USA (43.3%) [27].

In general, the prevalence of CKD increased with age within the same study population [19-21,23,27,29-31,34,37,40,43]. For example, the prevalence of CKD from the NHANES III study from 1988 to 1994 [21], a cross-sectional representative survey in USA, was 0.2% in the age group 20–39 years, 1.8% in the age group 40–59 years, 7.6% in the age group 60–69 years, and 24.9% in persons aged 70 and older as determined by means of the MDRD equation. Similar age-related increases of prevalence were observed in other studies as well, regardless which equation was used [19,20,23,27,29-31,37,40,43].

Coresh et al. [20] compared prevalence of CKD from two surveys of the National Health and Nutrition Examination Survey (NHANES III) conducted from 1998 to 1994 and 1999 to 2000 separately. They found that the overall prevalence of CKD was similar in both surveys (4.5% from 1998 to 1994, 3.8% from 1999 to 2000) and the proportions of CKD were also comparable with respect to gender and ethnicity.

#### Gender-specific prevalence of CKD

Most of the included studies also presented a gender-specific prevalence of CKD [19,21,23,24,27,30,31,34,36,40,42,43]. In general, the prevalence of CKD was greater in women than in men, regardless of age. A cross-sectional study reported a higher prevalence of CKD in Swiss women compared with men (4.5% in men and 11.5% in women) [31]. Brown et al. [19] reported remarkably high prevalences in both men and women, but women had a tendency to have a higher prevalence of CKD than men (14.4% in men and 16.2% in women, p = 0.09). Chadban et al. [36] reported a statistically significant gender-difference of prevalences derived from the AusDiab Study (9.3% in men and 13.0% in women, p = 0.002). In general, a higher prevalence of CKD in women compared with men was observed across age categories and also in various ethnic groups.

#### Ethnic-specific prevalence of CKD

In 3 studies from the USA, the prevalences of CKD were higher in Caucasians than in African Americans [20,21,27]. Notably, two studies from NHANES III reported CKD prevalences with respect to various ethnic subgroups: Mexican Americans were less likely to have CKD than African Americans and Caucasians [20,21]. The ethnic-specific prevalence was 49.9% in 11620 Caucasians and 33.7% in 8139 African Americans from the REGARDS cohort study [27].

Additionally, 6 studies from four countries in Asia (Thailand, China, Singapore and Japan) were conducted in comparable age groups [37-40,42,43]. Considering the estimating equations of GFR, a very high prevalence of CKD was found in a Chinese population (20.4%) [37] and a Japanese population (28.8%) [39] using the CG/BSA equation. When the MDRD equation was used, a lower prevalence was found (mean prevalence: 6.2%) as seen in other studies [37,38,40,42,43].

## Discussion

This systematic review summarized the prevalence of CKD in various population-based studies, which used a standardized definition of CKD and considered age-, gender- and ethnic-specific prevalence of CKD. Overall, the prevalence of CKD varied widely among the study populations and increased clearly with age. In general, females had a higher prevalence than males, especially in the middle aged groups. African Americans had a lower prevalence than Caucasians, and Asian populations had a relatively high prevalence. More importantly, the prevalence of CKD strongly depended on the estimating equations; the prevalence was much higher with the use of the CG/BSA equation than with the MDRD equation.

Although the burden of CKD seemed quite large in some studies, less than 2% of the CKD patients progresses to ESRD according to data from the US [44,45]. However, patients with early stages of CKD are clearly at risk for cardiovascular diseases [46]. Several interventions have been suggested to delay the progression of CKD or to prevent other complications of CKD, such as low-protein diets, strict control of blood pressure and proteinuria, smoking cessation, and the usage of lipid-lowering and anti-inflammatory medication [9].

### Prevalence of CKD and estimating equations

Serum creatinine concentration is the most commonly used biomarker to predict the level of kidney function, but it can be affected by various factors such as age, gender, ethnicity, muscle mass, dietary habit and specific drug use. The serum creatinine-based equations for GFR estimation overcome some of the limitations of using serum creatinine alone because they are adjusted for age, gender, ethnicity, or body size. So far, more than 25 serum creatinine-based equations for GFR estimation have been developed [47]. The MDRD equation and the CG/BSA equation are the most widely used equations for GFR estimation in practice; thus, we focus discussion on the performance of these two equations.

Lin et al. found that the MDRD equation was more precise and accurate to estimate GFR in healthy persons compared with the CG equation, but the MDRD equation consistently underestimated GFR and the CG equation overestimated GFR [48]. The latest study from Levey et al. calculated differences between estimated and measured GFR by stages of CKD [49], and reported that the MDRD equation was substantially better to estimate GFR in persons with CKD and with a measured GFR of less than 90 ml/min/1.73 m^2 ^compared to the CG/BSA equation. They also suggested that the use of a standard serum creatinine assay might improve the accuracy of GFR estimates. In contrast, a cross-sectional study of Veroort et al. reported a significant difference between measured GFR and estimated GFR after correcting by means of renal tubular secreted creatinine in healthy persons (9.0 ml/min/1.73 m^2 ^for CG/BSA equation, 10.7 ml/min/1.73 m^2 ^for the MDRD equation, p = 0.03) [50]. They concluded that the MDRD equation was less accurate in estimating GFR than the CG/BSA equation in persons with a normal GFR. However, the result should be carefully considered because of the small sample size of the study (n = 46). Meanwhile, Rule et al. showed a weak correlation between measured GFR and estimated GFR (r = 0.26, p < 0.001 for the MDRD equation; r = 0.35, p < 0.001 for the CG/BSA equation) in 365 healthy potential kidney donors [51], and they concluded that both equations were more or less inaccurate in healthy persons. Laboratory interferences and different study settings might partly explain the conflicting results.

A review article summarized the available data in 2005 and concluded that the MDRD equation provides a more accurate and clinical acceptable estimation of GFR than the CG equation in patients with GFR less than 60 ml/min/1.73 m^2 ^[47]. However, the CG/BSA equation is superior to the MDRD equation in terms of long time-period use in practice. Since the CG equation was published in 1976, it has been the most widely used equation in clinical practice, especially in drug dosage adjustments [14]. Later, it was adjusted to the body surface area (BSA) according to the Dubois and Dubois formula [17]. The adjustment for BSA had no affect on the accuracy of performance of the CG equation [48]. The MDRD equation was published in 1999 [15] and is widely used in clinical practice and epidemiologic studies [52]. Considering that these two equations were derived from patients with CKD, further studies should investigate for an improved prediction equation for GFR estimation in persons with normal or mildly decreased kidney function [53].

### Prevalence of CKD and personal characteristics

Age presents one of the most important factors that affect kidney function. Generally, kidney function is stable after infancy until late adulthood [54]. GFR declines by 1 ml/min/1.73 m^2 ^per year after the age of 30 years in healthy persons [5]. The decrease in kidney function might be due to the changes in the kidney structure associated with aging [55]. In the included studies, the elderly had a markedly higher prevalence of CKD and the prevalence increased with age in all populations, particularly among elderly persons aged 70 years or older. This steep increase in the prevalence of CKD in the elderly might be partly due to related comorbidities of CKD, such as cardiovascular diseases or diabetes. Moreover, the serum creatinine concentration remains within the normal range until a significant decrease of kidney function, especially in the elderly [56]. Serum creatinine is not a sensitive marker of GFR in older persons. In addition to the substantial effect of age on the kidney structure and kidney function, the same GFR level might have different pathophysiologic or non-pathophysiologic effects on kidney function in different age groups.

Furthermore, a gender-different prevalence of CKD was revealed in most included studies. Females had a higher prevalence of CKD than males. Females have less muscle mass as compared to males and the muscle mass is a major determinant of serum creatinine concentration [57]. Some risk factors for CKD in favour of males are unlikely to explain the difference between females and males, e.g. the prevalences of smoking and alcohol consumption, as well as the prevalence of cardiovascular diseases, which are generally higher in males than in females. The higher prevalence of CKD might partly be the result of an inaccurate correction factor for females in both equations. Additionally, the difference between females and males in glomerular structure, glomerular haemodynamics, and the hormone metabolism might play an important role in the gender disparity [58]. However, some uncertainties about the validity of prediction equations still remain, particularly when they are used in females.

### CKD in different ethnic groups

Both equations were developed from defined study populations. In the CG equation, no information on ethnicity was considered [14]. The MDRD equation was developed from study participants with only 12% of participants being African Americans [15,16]. In the US, the age-adjusted rate of ESRD for African Americans was almost 4 times higher than that for American Caucasians [59]. Li et al. concluded that the ethnical difference in the incidence of ESRD might partly be due to a higher prevalence of primary causal diseases of ESRD (e.g. diabetes, hypertension) and lower access to health care measures in African Americans as compared to American Caucasians [60]. McClellan et al. [27] investigated the ethnic difference of CKD in a population-based cohort of subjects aged 45 years or older and found that African Americans had a lower prevalence of CKD in early stages of CKD, but had a high prevalence of ESRD. They speculated that this disparity might be caused by different access to health care, poor control of other related risk factors, or differences in genetic factors, environmental exposures or life style. Studies hypothesized that the lower CKD prevalence in African Americans as compared to Caucasians might be due to impaired renal development and fewer nephrons resulting from a larger incidence of low birth weight in African Americans [61-63] or due to hyperfiltration and faster progression of CKD in African Americans [20,44]; it might also be the result of genetic differences, lifestyle difference, or different comorbidity, but the exact mechanisms remain unclear. Although the ethnical disparity could due to the different performance of GFR estimation equations in various ethnic groups, a recent study reported that the MDRD equation showed only little bias in African Americans [64].

We found unexpectedly higher prevalences of CKD when using the CG/BSA equation in Chinese and Japanese populations than in the US population. Interestingly, Chen et al. reported a large difference in the prevalence of CKD using the MDRD and the CG/BSA equations (2.5% using MDRD, 20.4% using CG/BSA) in the same study population from China [37]. Similar equation-differences were seen in 2 Japanese studies (10.3% using MDRD, 28.8% using CG/BSA) [39,42]. Zou et al. evaluated the validity of the MDRD and the CG/BSA equations among the Chinese population, and they suggested that further studies should be performed to determine the exact correction factor for CKD among the Chinese [65]. The original MDRD equation has been modified in China and Japan by specific coefficients [66-68]. A recent study in China developed a cystatin C-based equation for GFR estimation and combined serum creatinine and cystatin C measurement to improve accuracy of GFR estimation [69].

One of the included studies in the review assessed kidney function with cystatin C in elderly persons [35]. Cystatin C is under investigation as a promising marker for serum creatinine in estimating GFR [70,71]. More recent research has focused on the development of cystatin C-based equations for GFR estimation in the elderly and in different clinical setting such as in patients with diabetes and patients with cardiovascular disease et al. [72,73].

When looking on the results of our review the following limitations should be considered. Our primary interest was to identify all studies conducted in a population-based setting using a standard definition of CKD from the K/DOQI guideline to give an overview over the current situation of CKD worldwide. However, the differences in the prevalence of comorbidities and/or other factors related to risk of CKD, such as the prevalence of cardiovascular diseases, diabetes, infection, or lifestyle and socioeconomic factors, might affect the prevalence of CKD [74]. We were not able to take the underlying prevalence of these factors into account in the present review; further studies should be conducted and investigate them in more detail. It should be noted that the prevalence of CKD might be underestimated in the present review because other indications of kidney damage such as proteinuria were not considered by most of the included studies. In addition, the response quote should also be considered, which was only available in some of the studies. Reliable creatinine measurement in GFR is critical to the diagnosis of CKD. Different assays of creatinine measurement make it difficult to compare the prevalence of CKD across studies directly and having a worldwide standardization would provide a major improvement for the comparison of epidemiological studies.

## Conclusion

In conclusion, despite the disparities of GFR estimation, CKD is already a common disease in the worldwide general population. However, accurate detection of CKD in special subgroups remains inadequate. Besides putting more efforts to estimate GFR accurately in the general population, further studies should validate the means of GFR estimation in elderly persons, in females and in different ethnical groups. Additionally, the use of new emerging biomarkers like cystatin C should be an important issue in further studies of CKD.

## Competing interests

The author(s) declare that they have no competing interests.

## Authors' contributions

Q-LZ conducted the literature search, summarized all eligible papers, participated in synthesis of findings and drafted the manuscript. DR reviewed the manuscript and participated in synthesis of findings. Both authors read and approved the final manuscript.

## Pre-publication history

The pre-publication history for this paper can be accessed here:


